# Chitosan-salvianolic acid B coating on the surface of nickel-titanium alloy inhibits proliferation of smooth muscle cells and promote endothelialization

**DOI:** 10.3389/fbioe.2023.1300336

**Published:** 2023-11-13

**Authors:** Shijun Bi, Hao Lin, Kunyuan Zhu, Zechao Zhu, Wenxu Zhang, Xinyu Yang, Shanshan Chen, Jing Zhao, Meixia Liu, Pengyu Pan, Guobiao Liang

**Affiliations:** ^1^ Department of Neurosurgery, General Hospital of Northern Theater Command, Shenyang, China; ^2^ Graduate School, Dalian Medical University, Dalian, China; ^3^ Institute of Metal Research, Chinese Academy of Sciences, Shenyang, China; ^4^ Graduate School, China Medical University, Shenyang, China

**Keywords:** cerebrovascular disease, stent stenosis, interventional therapy, salvianolic acid B, chitosan, surface modification, endothelialization, restenosis

## Abstract

**Introduction:** Intracranial stents are of paramount importance in managing cerebrovascular disorders. Nevertheless, the currently employed drug-eluting stents, although effective in decreasing in-stent restenosis, might impede the re-endothelialization process within blood vessels, potentially leading to prolonged thrombosis development and restenosis over time.

**Methods:** This study aims to construct a multifunctional bioactive coating to enhance the biocompatibility of the stents. Salvianolic acid B (SALB), a bioactive compound extracted from Salvia miltiorrhiza, exhibits potential for improving cardiovascular health. We utilized dopamine as the base and adhered chitosan-coated SALB microspheres onto nickel-titanium alloy flat plates, resulting in a multifunctional drug coating.

**Results:** By encapsulating SALB within chitosan, the release period of SALB was effectively prolonged, as evidenced by the *in vitro* drug release curve showing sustained release over 28 days. The interaction between the drug coating and blood was examined through experiments on water contact angle, clotting time, and protein adsorption. Cellular experiments showed that the drug coating stimulates the proliferation, adhesion, and migration of human umbilical vein endothelial cells.

**Discussion:** These findings indicate its potential to promote re-endothelialization. In addition, the bioactive coating effectively suppressed smooth muscle cells proliferation, adhesion, and migration, potentially reducing the occurrence of neointimal hyperplasia and restenosis. These findings emphasize the exceptional biocompatibility of the newly developed bioactive coating and demonstrate its potential clinical application as an innovative strategy to improve stent therapy efficacy. Thus, this coating holds great promise for the treatment of cerebrovascular disease.

## 1 Introduction

Cerebrovascular disease, following ischemic heart disease, is the second major factor in global mortality and morbidity (Diseases and Injuries, 2020). In the treatment of cerebrovascular diseases, intracranial stents hold a pivotal position. Over time, stent technology has improved to include drug-eluting stents (DES) (Lee et al., 2018). DES involve the application of a pharmaceutical coating onto the surface of a metallic stent. After implantation, these stents gradually release drugs, thereby assisting in the inhibition of scar tissue formation around the stent and the maintenance of vascular patency (Lyu et al., 2020). Currently, widely utilized DES predominantly encompass rapamycin-eluting and paclitaxel-eluting stents (Sabate, 2022). Furthermore, bioactive stent coatings also have made rapid advancements, including polymer-coated stents, fibrin-coated metal stents, and phosphatidylcholine-coated stents. Polymer-coated stents have garnered significant attention due to their intrinsic elasticity and their capacity to reduce coagulation system activation (Chen et al., 2021). This feature results in a lowered risk of acute thrombus formation. Fibrin-coated metal stents have demonstrated the potential to promote endothelialization during peripheral vascular grafting and facilitate hemostasis during surgery (Link et al., 2020). This contributes to the preservation of the structural integrity of local blood vessels and a reduced risk of restenosis. Additionally, phosphorylcholine-coated stents have gained prominence owing to their hydrogel properties, which hinder protein adhesion, reduce thrombus formation, and enhance cellular biocompatibility (Liu et al., 2020). However, there are still significant limitations associated with the current approaches. When solely employing polymer coatings, experimental outcomes have not been as promising. Consequently, they are increasingly regarded as carriers for anti-thrombotic and anti-proliferative agents (Tzafriri et al., 2019). Additionally, it's worth noting that DES can potentially incite local tissue inflammation and immune responses (Choe et al., 2020). An even more critical concern is, although DES have reduced in-stent restenosis, a downside accompanies their use: the delayed re-endothelialization of the vascular wall (Qiu et al., 2022). Delayed re-endothelialization is a potential component that contributes to the establishment of late thrombosis and restenosis (Joner et al., 2006). Arterial healing after stent implantation is crucial in preventing stent-related complications, and the rebuilding of endothelial cells (ECs) has a substantial impact on recovery following stent insertion (Inoue et al., 2011). Stent coating design should shift its focus from individual targets to addressing issues such as delayed re-endothelialization, thrombosis, and restenosis. Additionally, stent coatings must possess high biocompatibility and multifunctionality to provide a favorable biological environment for re-endothelialization (Tahir et al., 2013). Therefore, our research aims to construct a novel coating with dual functionality: inhibiting smooth muscle cell proliferation while promoting endothelialization.

Salvianolic acid B (SALB), is well-known for its antioxidant activity (Katary et al., 2019). Furthermore, recent research has emphasized its anti-inflammatory and anti-apoptotic properties, which are of particular importance in the context of cardiovascular and cerebrovascular diseases (Xiao et al., 2020). In addition to the aforementioned functions, SALB serves a crucial dual purpose of inhibiting excessive proliferation of smooth muscle cells (SMCs), which is the primary cause of restenosis, and promoting angiogenesis, which is indicative of healthy vascular repair and regeneration (Ji et al., 2019; Hu et al., 2021b). This dual function of SALB aligns with the aim of enhancing stent biocompatibility, making SALB an ideal choice for improving vascular stents to withstand restenosis and promote vascular healing. Therefore, we hypothesize that SALB can be utilized to modify vascular stents to enhance their biocompatibility. Currently, the scientific literature only documents a limited amount of research on the use of SALB to modify the surface of vascular stents (Ji et al., 2019). The maximum blood concentration (Cmax) of SALB is approximately 910 μg/ml, and its half-life (t_1/2_) is approximately 105 min, which may not be sufficient for endothelial reconstruction (Wu et al., 2006). Chitosan has been demonstrated to be a drug delivery carriage and is increasingly being considered as a candidate material for tissue engineering (Khor and Lim, 2003). Chitosan demonstrates biodegradability, low toxicity, and antimicrobial activity, all of which are vital properties for implantable stents (Kim C. H. et al., 2018). Chitosan is frequently used as a carrier for sustained drug release in numerous routes of administration, such as oral, nasal, and mucosal delivery, aiming to achieve prolonged drug delivery (Mengatto et al., 2012). Our previous research on chitosan coating has shown that it has good biological activity and corrosion resistance (Eren et al., 2022). Additionally, the potential of chitosan to improve the biocompatibility of materials has been demonstrated (Venkatesan et al., 2014). This study optimized the surface of chitosan by utilizing its drug release function and its good biocompatibility and biodegradability (Abourehab et al., 2022). Therefore, our plan is to encapsulate chitosan on SALB to achieve the objective of prolonged drug release. Polydopamine (PDA) can essentially deposit on all types of organic and inorganic materials, forming functional coatings on their surfaces (Lee et al., 2007). To research the biological impacts of the stent on preventing restenosis and promoting endothelialization, nickel-titanium alloy flat plates were used in this experiment to imitate the stent. Therefore, our first step is to coat nickel-titanium alloy flat plates with a PDA layer. Subsequently, we will immobilize chitosan-encapsulated SALB microspheres onto the surface, thereby constructing a multifunctional drug coating.

To evaluate the promoting effect of this bioactive coating stent on endothelialization, we utilized methods such as biocompatibility experiments and cell experiments. This study aims to extend the current technology and enhance the material properties through an original approach.

## 2 Materials and methods

### 2.1 Materials

Salvianolic acid B (SALB) was acquired from Push Biotechnology (Chengdu, China). Dopamine hydrochloride, chitosan, and Crystal Violet Ammonium Oxalate Solution were acquired from Solarbio (Beijing, China). Nickel-titanium alloy flat plates were purchased from Yuyue Metal Products (Changzhou, China). Human umbilical vein endothelial cells (HUVECs) and smooth muscle cells (SMCs) were obtained from iCell Bioscience (Shanghai, China). Transwell chambers were procured from Nest Biotechnology (Wuxi, China).

### 2.2 Sample preparation

1 × 1 cm^2^ round nickel-titanium alloy flat plates were polished using sandpaper and then sequentially cleaned with acetone, anhydrous ethanol, and deionized water. A 2 mg/mL PDA solution was made by diluting dopamine hydrochloride in Tris-HCl buffer (10 mM, pH 8.5). The PDA solution was used to soak the nickel-titanium alloy samples, which were shaken for 24 h. To obtain nickel-titanium alloy samples coated with PDA, the samples were then washed with deionized water and allowed to air dry for 24 h.

The PDA-coated nickel-titanium alloy samples were submerged in a 2.0 mg/ml SALB solution at 4°C for 48 h. The samples were then air dried after being rinsed with PBS buffer solution. To create a solution containing 2.5 mg/ml of chitosan, add chitosan to an acetic acid solution and then raise the pH to 4.5. Stir the solution at room temperature until fully dissolved. Next, SALB was added to a solution of chitosan and stirred until complete dissolution. The final concentration of SALB was 2 mg/ml. Under high-speed magnetic stirring (4,000 rpm/min), a syringe was used to slowly add a 2 mg/ml solution of sodium tripolyphosphate (TPP) while stirring for a half-hour at room temperature. Finally, the chitosan-SALB nanoparticle solution underwent purification by passing through a membrane filter with a 0.22 μm micropore. The PDA-coated nickel-titanium alloy samples were then immersed in the purified chitosan-SALB nanoparticle solution at 4°C for 2 days. Following a thorough washing with PBS buffer solution, the samples were allowed to air-dry.

We used bare metal and dopamine-coated samples as the control group in all experiments to account for the potential biological reaction of dopamine. “BARE” refers to the unmodified nickel-titanium alloy flat plates, “PDA” refers to the single PDA coating, “SALB” refers to the coating of SALB on the PDA layer, and “CS-SALB” refers to the coating of chitosan-coated SALB on the PDA layer.

### 2.3 Surface characteristics

Scanning electron microscopy (SEM) (Quanta 450 FEG, FEI, Hillsboro, United States) was used to examine the nickel-titanium alloy plates’ coated surfaces with nanoparticles. The water contact angle of the nickel-titanium alloy plates’ surface was characterized using a water contact angle measurement device. The nickel-titanium alloy samples were fixed on the sample stage, and a droplet of distilled water was placed on the surface. After 2 s, the water contact angle was measured. Using ImageJ software, the samples’ water contact angles at the surface were examined.

### 2.4 Drug release *in vitro*

The SALB and CS-SALB group samples were immersed in test tubes containing 3 ml of PBS solution and continuously shaken at 37°C and 60 rpm. 1 ml of the solution was taken out of the test tubes at predetermined intervals, and an equivalent volume of PBS solution was added to keep the total volume constant. Samples were collected at specific intervals: 6, 12, 18, 24, and 36 h, and 2, 4, 7, 14, and 28 days. The collected solution from the test tubes was subjected to ultrasound treatment, followed by filtration, and a microplate reader (Epoch, Biotek, Winooski, United States) was used to measure the absorbance at 280 nm. The release standard curve of SALB was constructed, and the release amount was calculated from the release absorbance value.

### 2.5 Hemocompatibility experiments

The automated blood coagulation analyzer (CS-5100, Sysmex, Kobe, Japan) conducted tests for activated partial thromboplastin time (APTT) and prothrombin time (PT). After soaking each set of samples in PBS solution for an hour, 500 μl of platelet-poor plasma were added, followed by a 30-min incubation at 37°C. APTT and PT tests were then conducted to analyze the clotting time of the samples.

The samples were submerged in a solution of PBS containing BSA (1 mg/ml) for 1 ml, and then incubated for 1 h at 37°C to determine the quantity of adsorbed protein. The difference in concentration of the BSA solution before and after immersion is used to calculate the adsorbed protein.

### 2.6 HUVECs and SMCs proliferation assay

HUVECs and SMCs were grown in DMEM with 10% FBS supplement and incubated at 37°C in a 5% CO_2_ incubator (BC-J160S, Boxun, Shanghai, China) for the duration of the experiment. 24-well culture plates were used to hold the samples. Each well was then filled with a 1 mL solution of HUVECs or SMCs (2 × 10^3^ cells/mL). The plates were incubated for 1, 3, and 5 days, respectively, at 37°C and 5% CO_2_. After the allotted incubation time had passed, the culture media was carefully removed, and each well received 1 mL of DMEM with 100 μL of Cell Counting Kit-8 (CCK-8). After 2 h of incubation at 37°C, the samples were examined to determine the number of cells in each group by measuring the optical density (OD) at 450 nm with a microplate reader.

### 2.7 HUVECs and SMCs migration assay

The groups of samples underwent sterilization using ethylene oxide and were then immersed in 2 mL of DMEM for 72 h to obtain the respective extracts. HUVECs or SMCs were infused into 6-well plates at a density of 5 × 10^5^ cells/ml and cultivated in 10% FBS DMEM until a monolayer of cells formed. A 200 μL pipette tip was used to scrape the cell layer’s surface in order to produce a cell-free area. Subsequently, 10% FBS DMEM was changed to serum-free DMEM that included extracts from each sample group. The migration of cells in the scratched region was seen under a light microscope (CKX-53, Olympus, Tokyo, Japan) at the appropriate time intervals following a 24-h incubation period at 37°C with 5% CO_2_. The ImageJ software was used to analyze each collection of scratches.

The transwell chamber was used to analyze the effect of each set of samples on the migration of HUVECs and SMCs. First, the samples were placed in the lower chamber of the transwell chamber. Then, serum-free DMEM culture medium containing HUVECs or SMCs (200 μL, 1.0 × 10^5^ cells/mL) was added to the upper chamber. The culture medium was taken out after 24 h of incubation. After using a cotton swab to clean the upper chamber and washing it with PBS, the cells were fixed for 30 min with 4% paraformaldehyde. After PBS-rinsing, 0.1% crystal violet solution was used to stain the cells. Cell migration to the lower chamber was observed under the microscope (CKX-53, Olympus, Tokyo, Japan). The cell numbers migrating to the lower chamber of the transwell chamber were counted using ImageJ software.

### 2.8 HUVECs and SMCs adhesion assay

A suspension of HUVECs (1 × 10^4^ cells/mL) or SMCs (2 × 10^4^ cells/mL) was placed in each well of the 24-well plates containing the samples. The plates were kept in a 37°C, 5% CO_2_ incubator. After the medium had been removed after 3 days, the samples had been cleaned with PBS, and the cells had been fixed with 4% paraformaldehyde for 30 min. Each well was added to an antifade mounting medium containing DAPI stain and incubated for 5 min in the dark. Cell adhesion levels were assessed by observing the cells under a fluorescence microscope (BX-53, Olympus, Tokyo, Japan).

The cell culture media was taken out of the well plates and PBS-washed after 3 days of incubation. Following that, the samples from each group were fixed for 30 min in 4% paraformaldehyde. Afterwards, PBS was used to rinse the samples. Triton X-100 0.2% solution was used to permeabilize cells for 15 min at 4°C. The samples were then treated with 1% BSA for an hour at 37°C. Furthermore, the cells were stained with 1% fluorescently labeled phalloidin for 60 min at room temperature, protected from light, followed by washing with PBS. Incubate the sample in the dark with antifade mounting medium containing DAPI for 5 min. Finally, using fluorescence microscopy (BX-53, Olympus, Tokyo, Japan), the cell morphology of the sample is analyzed and photographed.

### 2.9 Statistical analysis

Version 9.5.0 of GraphPad Prism for Windows (GraphPad Software) was used to conduct the statistical analysis. All data are presented as mean ± standard deviation (SD). The two-way ANOVA and one-way ANOVA with Tukey *post hoc* comparison were used to compare the results. The statistical significance level was determined using a minimum sample size of three. Values of *p* < 0.05 were considered significant.

## 3 Results

### 3.1 Surface characteristics of nickel-titanium alloy samples

**FIGURE 1:**
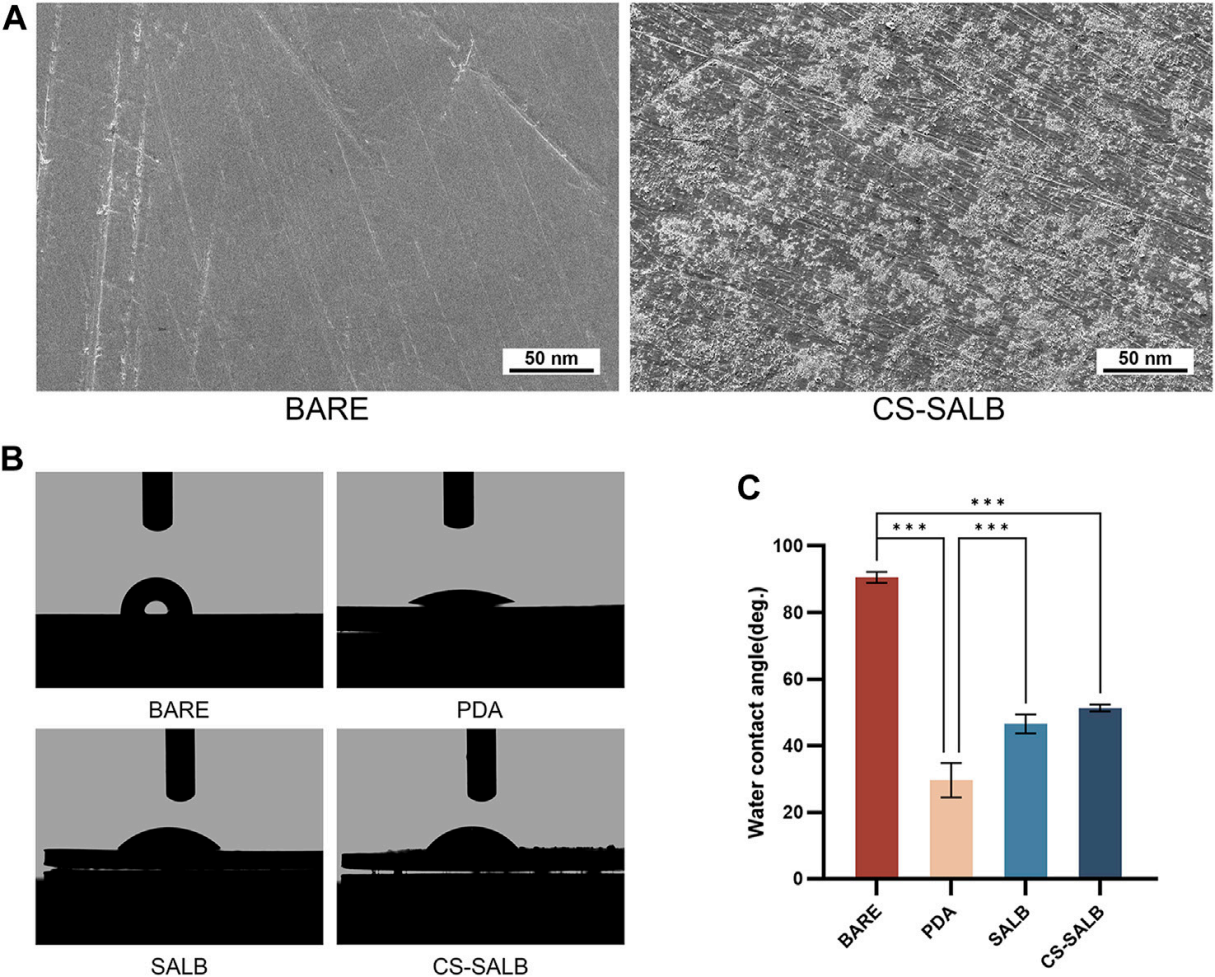
SEM and water contact angle analysis. **(A)** Scanning electron microscopy (SEM) images comparing the BARE group and CS-SALB group. **(B)** Representative images of water contact angle testing on different surfaces. **(C)** Quantification of water contact angles on different surfaces. Significance is indicated by asterisks: *p < 0.05, **p < 0.01, ***p < 0.001.

The nickel-titanium alloy plates’ surface morphology was examined using SEM (Figure 1A). The SEM images revealed a relatively smooth surface of the nickel-titanium plates with only fine polishing lines. After fixation, the SALB nanospheres were uniformly adhered to the surface, presenting spherical shapes with a uniform particle size distribution. Water contact angle measurements were used to evaluate the hydrophilicity of the material’s surface. Smaller contact angles indicate better hydrophilicity. Figure 1B shows the images of water contact angle testing for each group, and Figure 1C presents the water contact angle values for the BARE group (90.57° ± 1.32°), PDA group (29.67° ± 4.21°), SALB group (46.57° ± 2.29°), and CS-SALB group (51.33° ± 0.83°). The PDA group exhibited the best hydrophilicity, while the CS-SALB group showed significantly enhanced hydrophilicity compared to the BARE group. The SALB group’s and the CS-SALB group’s levels of hydrophilicity were not significantly different.

### 3.2 *In vitro* drug release

**FIGURE 2:**
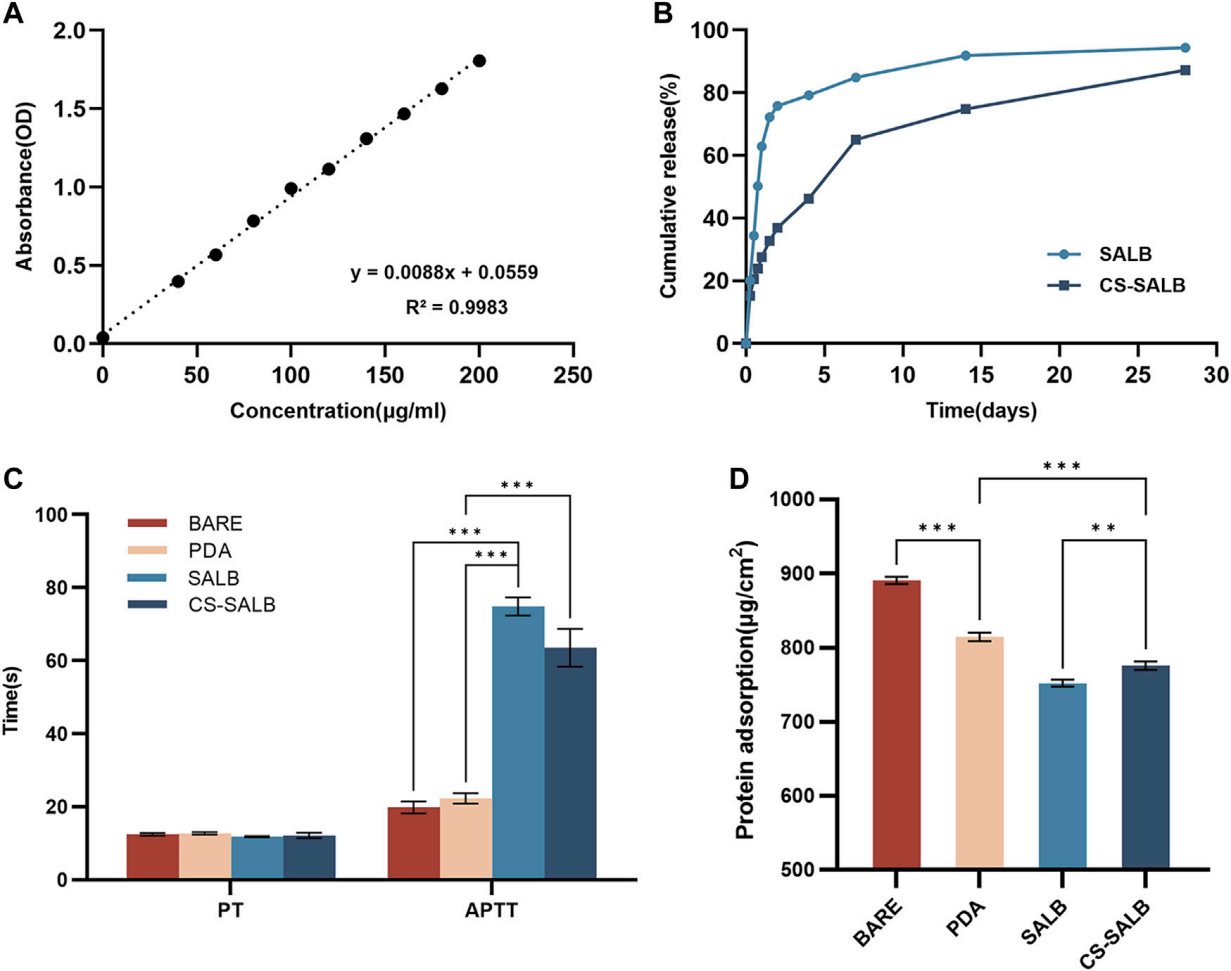
*In vitro* drug release and hemocompatibility experiments. **(A)** Standard curve showing the concentration of salvianolic acid B (SALB) drug. **(B)** Drug release curves of the SALB and CS-SALB group. **(C)** Activated partial thromboplastin time (APTT) and prothrombin time (PT) measurements. **(D)** Protein adsorption onto the surfaces of each group. Significance is indicated by asterisks: *p < 0.05, **p < 0.01, ***p < 0.001.

A standard curve of SALB concentration was generated using GraphPad Prism software for linear fitting (Figure 2A). The amount of SALB released from the drug coating on the sample’s surface during the duration of the experiment was calculated by contrasting the absorbance values to the standard curve. The drug-loaded microspheres released the drug over time, as shown by the *in vitro* drug release behavior. The drug release curves of the CS-SALB group are shown in Figure 2B. After the rapid release during the first 2 days, the release rate of SALB slowed significantly with increasing release time. More than 80% of SALB was released cumulatively within a period of 28 days.

### 3.3 Hemocompatibility experiments

The results of coagulation function tests conducted on the surface of each sample group are presented in Figure 2C. There was no statistically significant difference in PT between the samples from any group. However, APTT was significantly prolonged in the SALB and CS-SALB groups. Figure 2D shows the amount of protein adsorbed onto the surfaces of the samples, with values for the BARE group (890.92 ± 3.94), PDA group (814.66 ± 4.71), SALB group (752.27 ± 3.77), and CS-SALB group (775.82 ± 4.47). The CS-SALB group showed significantly lower protein adsorption, indicating better blood compatibility.

### 3.4 Effects of nickel-titanium alloy plates on HUVECs and SMCs

**FIGURE 3:**
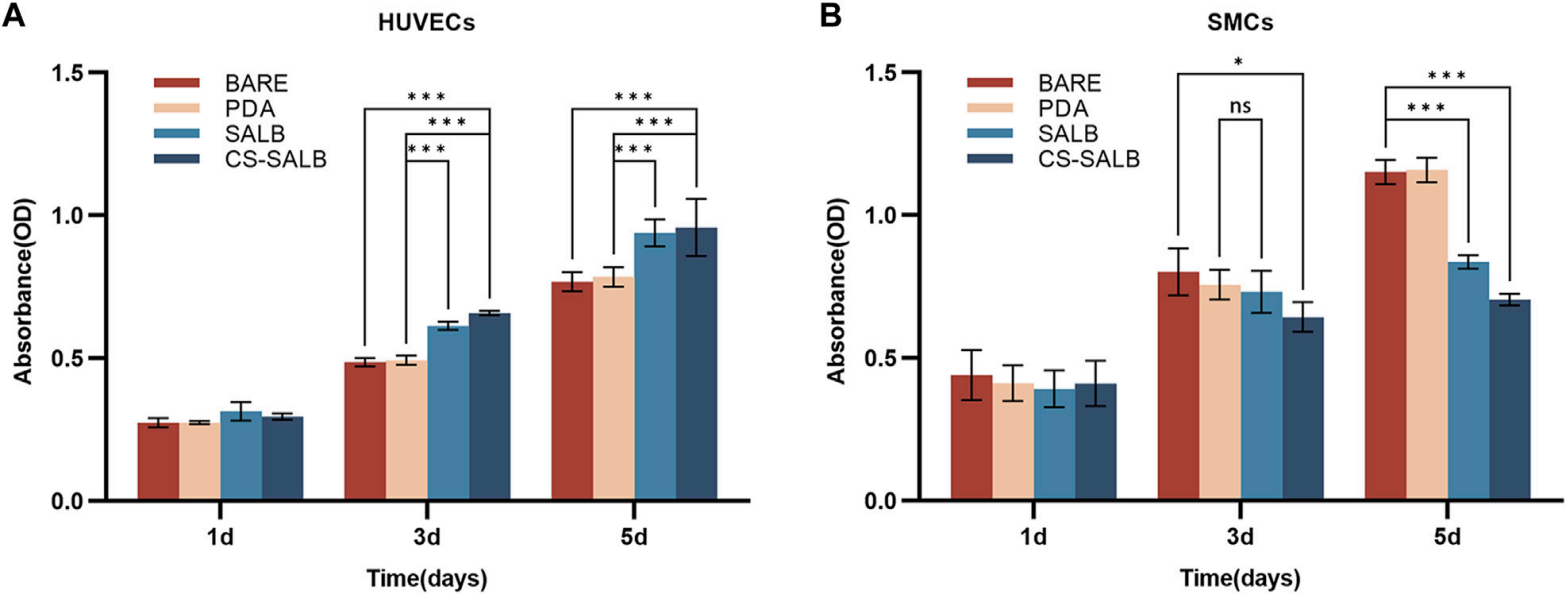
Cell viability assessed by CCK-8 assay. **(A)** Viability of HUVECs at 1, 3, and 5 days as determined by CCK-8 assay. **(B)** Viability of SMCs at 1, 3, and 5 days as determined by CCK-8 assay. Significance is indicated by asterisks: *p < 0.05, **p < 0.01, ***p < 0.001; ns means no statistical difference.

The effect of each group of samples on cell proliferation is shown in Figures 3A, B. All samples showed continuous proliferation of cells on their surfaces during the 5-day incubation period. After 1 day of culture, there were no significant variations in the proliferation of HUVECs and SMCs across groups. On days 3 and 5, the SALB and CS-SALB groups’ surfaces had a much greater rate of HUVECs proliferation than the BARE and PDA groups’ surfaces. On day 5, there was no discernible difference between the SALB and CS-SALB groups in the pace at which HUVECs proliferated. After the 3-day cultural phase, the CS-SALB group exhibited a higher inhibitory effect on SMCs proliferation than the BARE group, while no significant differences were observed for the remaining groups. After the 5-day culturing period, the proliferation rates of SMCs in the SALB and CS-SALB groups were not significantly different, but both groups showed significant inhibition of SMCs proliferation compared to the BARE and PDA groups.

**FIGURE 4:**
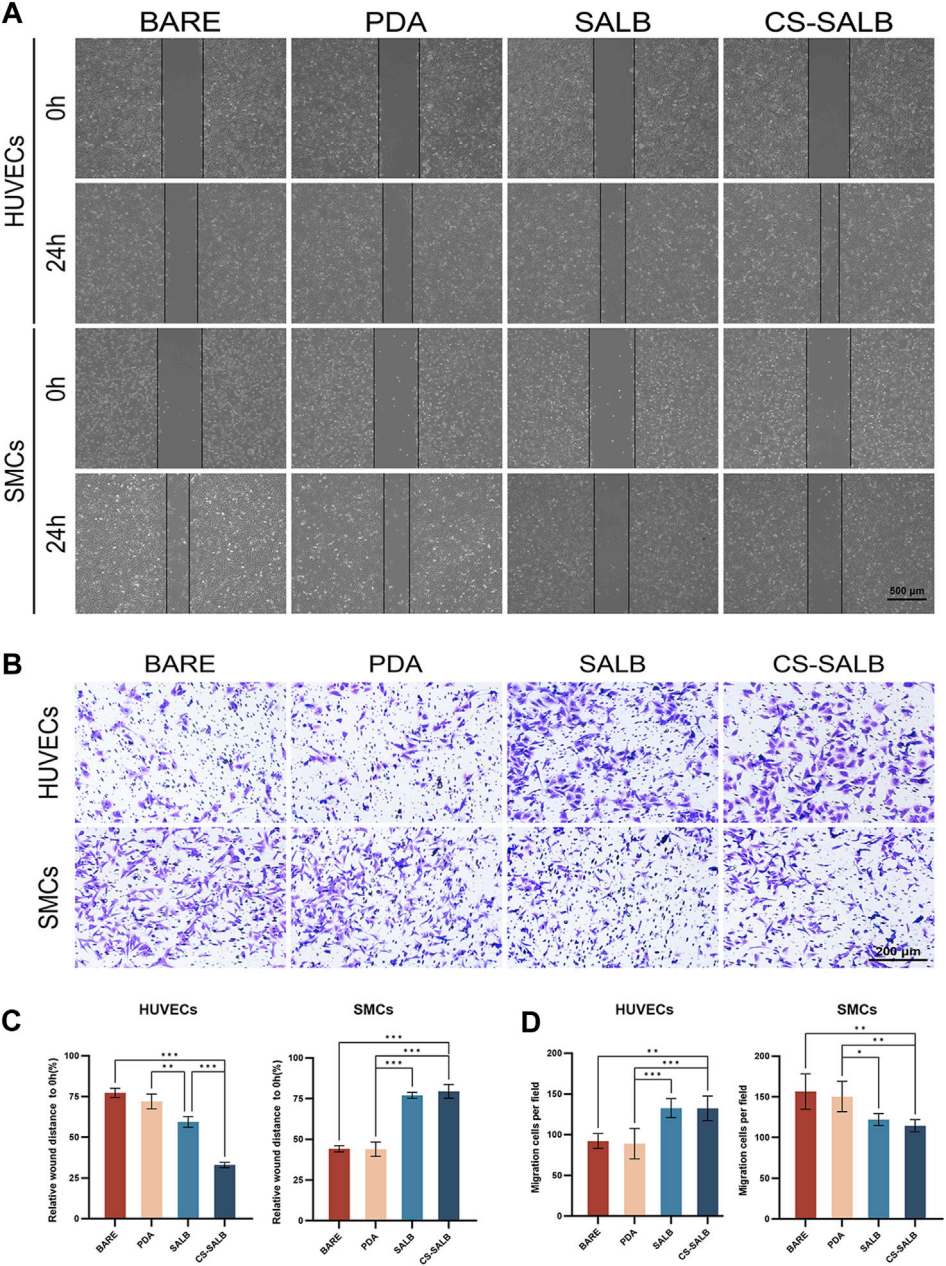
Cell migration capacity assessed by scratch assay and Transwell assay. **(A)** Impact of extracted eluates from each group after 3-day immersion on the migration of HUVECs and SMCs as measured by scratch assay. **(B)** Influence of co-culturing with each group’s samples for 24 h on the invasiveness of HUVECs and SMCs as determined by Transwell assay. **(C)** Quantitative assessment of the effects of eluates from each group on the migration of HUVECs and SMCs. **(D)** Quantitative evaluation of the effects of eluates from each group on the invasiveness of HUVECs and SMCs. Significance is indicated by asterisks: *p < 0.05, **p < 0.01, ***p < 0.001.

Figures 4A, B shows the effect of each sample group on cell migration ability. After 24 h, the results for HUVECs showed no apparent distinction between the BARE and PDA groups. However, the number of migratory cells was greater in the SALB group compared to the BARE group and noticeably higher in the CS-SALB group compared to the SALB group. The number of migrating SMCs between the BARE group and the PDA group after 24 h of culture did not differ significantly. However, the CS-SALB group significantly inhibited the migration of SMCs compared with the BARE group (Figure 4C). After 24 h, there was no discernible difference between the SALB and CS-SALB groups in the quantity of HUVECs migrating to the lower chamber. In contrast to the BARE and PDA groups, both groups had a much higher number of migrating cells. Both the SALB and CS-SALB groups significantly inhibited the migration of SMCs from the upper to the lower chamber (Figure 4D).

**FIGURE 5:**
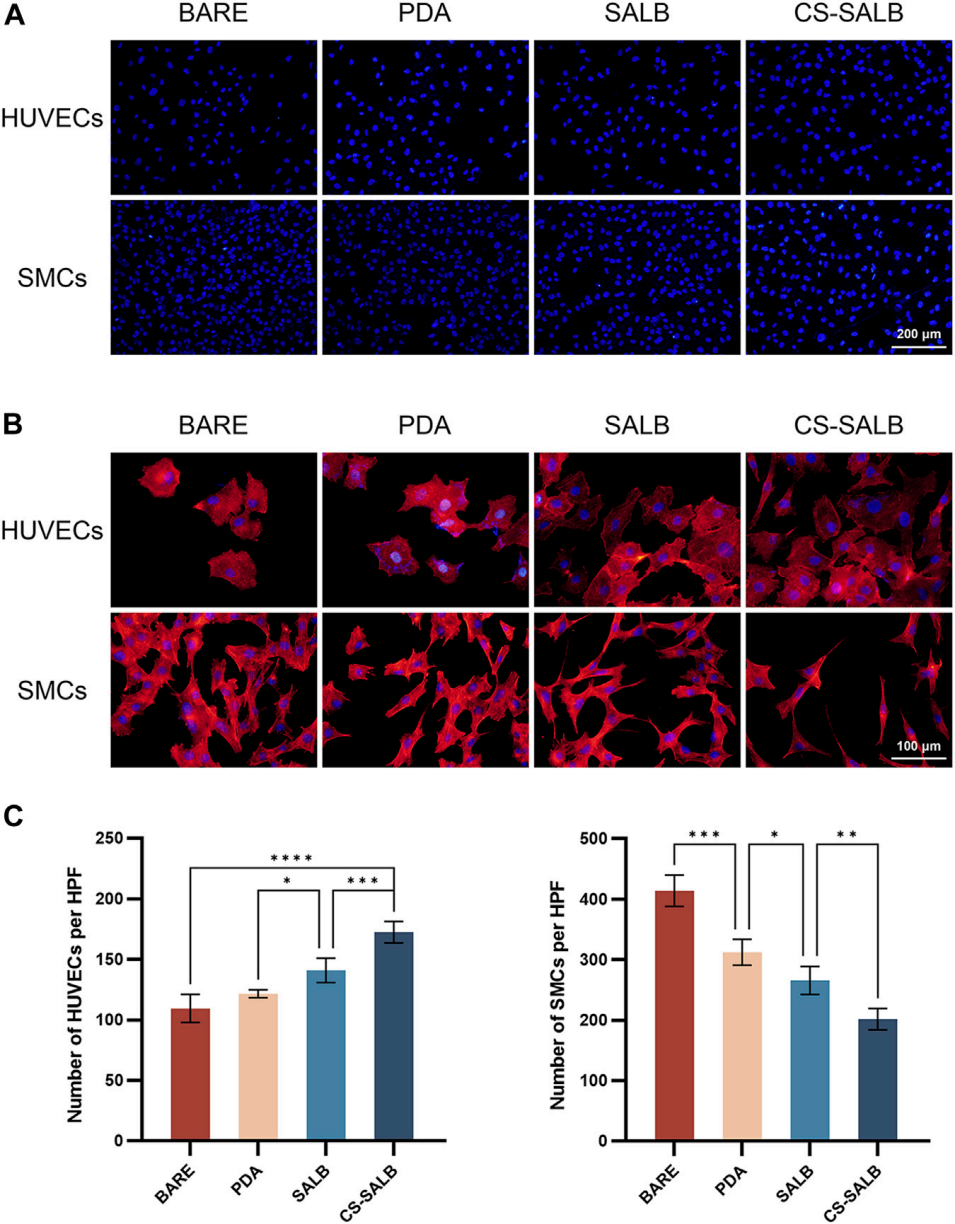
Cell adhesion capacity and cell morphology. **(A)** DAPI staining of HUVECs and SMCs attached to the surface of the samples. **(B)** Fluorescence images showing the adhesion of HUVECs and SMCs on the surface of the samples. Actin filaments are stained in red, and cell nuclei are stained in blue. **(C)** Quantification of cell proliferation by counting the number of DAPI-positive cells. Significance is indicated by asterisks: *p < 0.05, **p < 0.01, ***p < 0.001.

As shown in Figure 5A, the cell growth was visualized by DAPI staining. The morphology of adherent cells in each sample group is depicted in Figure 5B. HUVECs displayed polygonal morphology on all surfaces. SMCs adhered well to the surfaces of the BARE group, PDA group, and SALB group. Conversely, SMCs in the CS-SALB group exhibited spindle-like narrowing. After 3 days, the SALB group and the CS-SALB group demonstrated significantly increased numbers of adherent HUVECs. Comparing the CS-SALB group to the other groups, the number of adherent SMCs dramatically decreased (Figure 5C).

## 4 Discussion

The research results demonstrate that the SALB coating surface on nickel-titanium alloy exhibits excellent biocompatibility and can effectively promote HUVECs proliferation, adhesion, and migration. Additionally, they can inhibit SMCs proliferation, adhesion, and migration. These findings suggest that the developed bioactive coating holds potential for promoting endothelialization and preventing restenosis.

ECs and SMCs are involved in vascular reconstruction following stent placement (Jin et al., 2019). ECs are responsible for maintaining vascular function, while SMCs regulate vascular contraction and expansion, which jointly influence the biocompatibility and functionality of stents (Chen et al., 2021). Diaz-Rodriguez discovered that the coating of CD31-mimetic peptide significantly reduced the activation of platelets and leukocytes *in vitro*, which proved advantageous to the growth of physiological ECs on the scaffold, thus accelerating the process of arterial wall healing (Diaz-Rodriguez et al., 2021). Exosome-eluting stents have been found to accelerate vascular healing, promote the formation of ECs, reduce restenosis, regulate macrophage polarization, minimize inflammation, and promote tissue repair after ischemic injury (Hu et al., 2021a). Moreover, a bionic modification method was successfully developed to treat the surface of degradable magnesium alloys by micropatterning the natural extracellular matrix (ECM) secreted by SMCs and ECs. The bionic ECM coating improves the endothelialization of the material’s surface and exhibits better blood compatibility as well as anti-proliferation and anti-inflammatory effects (Liu et al., 2022). To summarize, drug coating can enhance vascular healing and repair by encouraging the creation of ECs or inhibiting the proliferation of SMCs. To achieve better results in specific clinical applications, further exploration and optimization of the coating’s composition and characteristics are necessary.

At present, a lot of stent coating studies focus on a singular biological process, either preventing restenosis or promoting re-endothelialization (Hu et al., 2021a; Diaz-Rodriguez et al., 2021). However, stents with dual functions of re-endothelialization and inhibiting SMCs proliferation show a high antithrombotic effect, rapid endothelialization, and long-term prevention of restenosis *in vivo* (Lyu et al., 2020). Consequently, it is highly significant to develop a drug coating that serves dual functions to enhance vascular reconstruction (Alexander et al., 2018). SALB, a phenolic carboxylic acid extracted from the water extract of Salvia miltiorrhiza, has been proven to possess two bioactivities: inhibiting SMCs proliferation and promoting angiogenesis (Lay et al., 2003; Pan et al., 2012). Consequently, our research endeavors to investigate a novel coating strategy, utilizing SALB as the fundamental element, to concurrently promote endothelialization and impede the proliferation of SMCs during metal surface modification to achieve overall better stent implanting performance. Nevertheless, one major challenge encountered at the beginning of the experiment was the rapid release of SALB *in vivo*. To overcome this issue, we developed a drug coating that could control the sustained release of SALB to leverage its biological effects on blood vessels. The use of chitosan as a nanocarrier system provides the advantages of sustained release, biodegradability, and modifiability (Cheng and Dai, 2022). For these reasons, chitosan is commonly employed in drug-sustained release systems to achieve prolonged drug release (Wu et al., 2006).

The analysis of the drug release curve data shows two distinct phases consisting of an initial burst phase and a sustained release phase (Figure 2B). During the first 24 h, the initial burst phase of the drug release curve exhibits a significant increase in the release rate, followed by a transition to the sustained release phase, where the release rate gradually decreases over the next 36 h to 28 days and eventually stabilizes. The pronounced rapid release of the drug during the initial phase can be attributed to its physical adsorption onto the surface of the chitosan coating. Over time, the depletion of physically adsorbed drugs causes a gradual decline in the release rate, the stable biodegradability of chitosan mainly affects the slow-release stage (Bruinsmann et al., 2019). TPP is a commonly employed ionic crosslinking agent (Abdelgawad and Hudson, 2019). The positively charged R-NH3+ groups of chitosan engage with the negatively charged oxygen molecules of TPP’s phosphate groups (R'-O-), facilitating proton exchange with the chitosan amino groups through electrostatic interactions (Saeedi et al., 2022). Physical crosslinking eliminates the need for chemical crosslinking agents and emulsifiers, which can be harmful to medications (Berger et al., 2004). SALB diffuses into the chitosan matrix through this ionic crosslinking reaction (Fan et al., 2012). Sustained release of SALB occurs during the degradation of chitosan particles, allowing the continuous diffusion of the drug into the surrounding environment.

PDA served as the interface between chitosan-SALB drug-loaded microspheres and the material surface in this study. PDA is a novel polymer known for its remarkable adhesive properties, making it suitable for deposition on both hydrophilic and hydrophobic surfaces. The polymerization process relies on the oxidative polymerization of dopamine under alkaline conditions (Mrowczynski et al., 2018). PDA-based coatings not only improve the solution stability of polymer nanoparticles but also provide an active platform for secondary reactions, enabling the immobilization of more functional components. This offers an opportunity for further improvement of material performance and functionality (Liu et al., 2016). PDA shows excellent biocompatibility due to its status as an important component of natural melanin widely present in the human body (Li et al., 2021). Moreover, it can decrease the frequency of unfavorable responses caused by introducing exogenous materials (Liu et al., 2014; Marinas et al., 2020).

Clear polishing marks could be seen on the BARE group’s surface using SEM. However, in the CS-SALB group, the nanoparticles formed uniform clusters that successfully adhered to the PDA coating surface. The chitosan microspheres loaded with SALB were attached to the PDA coating surface through both physical adsorption and chemical cross-linking, effectively preventing the detachment of drug-loaded microspheres from the coating surface. A comparison of the water contact angles on the surfaces of the different groups showed differences in surface hydrophilicity. The contact angle significantly decreased in the PDA group, and after the fixation of CS-SALB microspheres on the surface, the contact angle slightly increased. Past research has thoroughly illustrated PDA’s superhydrophilicity, the material can rapidly absorb water after being treated with a PDA coating, and this treatment causes the material’s superhydrophilicity to endure for a minimum of 28 days (Kopec et al., 2020). It has been reported that materials having water contact angles within the range of 40°–70° are conducive to cell adhesion (He et al., 2022). The contact angle of the CS-SALB group exceeds that of the BARE group, facilitating cell adhesion. This shows that the sample surface’s hydrophilicity is improved by the application of the chitosan-SALB coating.

Placement of the stent within the blood vessel leads to biocompatibility issues caused by its foreign body properties (Zhu et al., 2022). To tackle this issue, experiments related to blood compatibility were conducted. Once the stent comes into contact with blood, blood proteins adhere to the stent’s surface, causing a protein layer to develop. One of the blood proteins, fibrinogen, may cause platelet activation, aggregation, and the start of the coagulation cascade (Rubic et al., 2022). BSA has been widely used in biomedical research due to its comparability and validation with existing literature and findings (Ribeiro et al., 2022). BSA exhibits several favorable properties, including biocompatibility and solution stability, when compared to HSA and other proteins (Sangra et al., 2017). Therefore, BSA is a suitable choice for modeling the interaction between blood and stents, so we chose BSA for the protein adsorption assay. The results of the protein adsorption test indicate that the chitosan-SALB coating significantly decreases the protein adsorption rate on the material’s surface, with potential implications for reducing the incidence of thrombosis. The evaluation of coagulation time revealed that the CS-SALB group had a significant prolongation of APTT. Our results suggest that surface loading of materials with PDA, chitosan, and SALB coatings can improve biocompatibility and reduce coagulation activity, thereby potentially reducing thrombosis risk.

We carried out pertinent studies by culturing HUVECs and SMCs on the samples in order to assess the *in vitro* bioactivity of the coatings. We assessed cell proliferation activity using the CCK-8 assay, cell migration ability through scratch and transwell assays, and cell adhesion quantity and morphology through cell adhesion experiments.

While our study demonstrated its potential dual role in regulating both ECs and SMCs, further investigation is needed to understand the precise molecular mechanisms involved. Previous studies have shed light on the potential mechanisms underlying SALB’s actions. SALB has been shown to increase the expression of phosphorylated STAT3 and VEGF signaling pathway-related proteins, upregulate the genes for VEGF and its receptors, and stimulate angiogenesis (Lay et al., 2003; Kim R. et al., 2018; Wang et al., 2018). Our *in vitro* research demonstrated that the SALB drug coating significantly affected the proliferative and migratory capacities of HUVECs. These findings indicate that drug coating may be a significant factor in encouraging re-endothelialization. In terms of SALB’s effect on SMCs, it has been discovered that SALB can regulate cell cycle regulators and miR-146a, as well as inhibit the CXCR4 signal pathway molecule’s expression level, thereby preventing the proliferation and migration of SMCs (Pan et al., 2012; Lee et al., 2014; Zhao et al., 2019). Our observation indicates that the SALB drug coating can potentially impede the proliferation of SMCs and reduce stenosis. Further study on the molecular biology mechanism of SALB on ECs and SMCs is needed.

In certain experiments, the SALB group exhibited slightly superior performance to the CS-SALB group. This could be explained by the burst release of the drug coating in the SALB group resulting from the lack of chitosan, leading to improved short-term effects. Long-term drug release from the coating is especially important in addressing the issue of late complications and restenosis after stent implantation. Based on relatively long-term experimental results, the CS-SALB group demonstrated similar or even better functional effects compared to the SALB group in this study.

Our focus was on exploring the potential of SALB in the field of vascular stents, where we conducted relevant experiments with only two cell types that are important for vascular reconstruction. To validate the effects of this novel coating, further long-term animal experiments are needed. Furthermore, for simulating the stent material, we used nickel-titanium alloy flat plates in this study. An investigation using the controlled and uniform coating of this formulation onto commercially available stents will be helpful to prove our opinion.

## 5 Conclusion

The combination of chitosan and SALB in the coating improved the biocompatibility of the bare metal surface. And those may promote the adhesion and migration of ECs while inhibiting the proliferation and migration of SMCs. This composite coating might have the potential to prevent stenosis in the field of vascular interventions.

## Data Availability

The original contributions presented in the study are included in the article/Supplementary Material, further inquiries can be directed to the corresponding authors.
